# An Improved Optimization Function to Integrate the User’s Comfort Perception into a Smart Home Controller Based on Particle Swarm Optimization and Fuzzy Logic

**DOI:** 10.3390/s23063021

**Published:** 2023-03-10

**Authors:** Jonatha Rodrigues da Costa, Giovanni Cordeiro Barroso, Darielson Araújo de Souza, Josias Guimarães Batista, Antonio Barbosa de Souza Junior, Clauson Sales do Nascimento Rios, Felipe José de Sousa Vasconcelos, José Nogueira do Nascimento Júnior, Ismael de Souza Bezerra, Alanio Ferreira de Lima, Killdary Aguiar de Santana, José Raimundo de Oliveira Júnior

**Affiliations:** 1 Department of Electrical Engineering, Federal University of Ceará, Fortaleza 60455-760, CE, Brazil; 2 Department of Computing, Federal Institute of Ceará—IFCE Campus Maracanaú, Maracanaú 61925-315, CE, Brazil; 3 Department of Industry, Federal Institute of Ceará—IFCE Campus Fortaleza, Fortaleza 60040-215, CE, Brazil; 4 Department of Industry, Federal Institute of Ceará—IFCE Campus Maracanaú, Maracanaú 61925-315, CE, Brazil

**Keywords:** fuzzy logic, load-side management, particle swarm optimization, smart grids, smart home controllers

## Abstract

Scheduling residential loads for financial savings and user comfort may be performed by smart home controllers (SHCs). For this purpose, the electricity utility’s tariff variation costs, the lowest tariff cost schedules, the user’s preferences, and the level of comfort that each load may add to the household user are examined. However, the user’s comfort modeling, found in the literature, does not take into account the user’s comfort perceptions, and only uses the user-defined preferences for load on-time when it is registered in the SHC. The user’s comfort perceptions are dynamic and fluctuating, while the comfort preferences are fixed. Therefore, this paper proposes the modeling of a comfort function that takes into account the user’s perceptions using fuzzy logic. The proposed function is integrated into an SHC that uses PSO for scheduling residential loads, and aims at economy and user comfort as multiple objectives. The analysis and validation of the proposed function includes different scenarios related to economy–comfort, load shifting, consideration of energy tariffs, user preferences, and user perceptions. The results show that it is more beneficial to use the proposed comfort function method only when the user requires SHC to prioritize comfort at the expense of financial savings. Otherwise, it is more beneficial to use a comfort function that only considers the user’s comfort preferences and not their perceptions.

## 1. Introduction

In the context of DR, measures to improve the energy chain unfold in two areas: SSM and DSM. The first area aims to improve the generation, transmission, and distribution of electricity, while the second area aims to influence the consumption profile of households through flexible tariffs or energy bonuses [1,2]. In this scenario, residential consumers can use SHC to take advantage of benefits offered by the utility [3]. These controllers may incorporate residential comfort functions [4,5,6], residential load modeling, the ability to manage DG associated with ESS and EV, and a collaborative/non-collaborative relationship between prosumers [7].

Specifically in the context of DSM, residential load modeling and user comfort modeling are presented as one of the important measures that fulfill the above idea. The modeling of residential loads is based on the successive measurement of electrical quantities associated with residential loads connected to the electrical power grid. On the other hand, user comfort modeling comprises both psychological and physiological issues. Psychological issues are related to the perceptions of human beings regarding their way of feeling and responding to stimuli felt by the human body [8,9]. This perception can affect an individual’s mood, emotional state, and satisfaction to the point of making them uncomfortable in an environment that is pleasant for others, or vice versa [10,11,12]. Physiological aspects, on the other hand, are related to well-being and safety, including thermal comfort, visual comfort, and ambient air quality [13,14,15]. In this context, the modeling of residential loads and user comfort may be characterized by disjoint metrics resulting from different indices and by parameters separated from each other.

However, for a multi-objective SHC system, residential loads can have their own comfort index that measures overall comfort associated with load scheduling and user preferences. Thus, it is reasonable to state that there is a gap in the SHC state-of-the-art in relation to the incorporation of the user’s perception of residential comfort, as the algorithms proposed for managing residential loads associated with SHC do not consider the user’s perceptions of comfort, only their preferences. Therefore, an SHC in this configuration can present comfort indices that do not correspond to the user’s real perception of comfort, because users’ psychological factors can influence their perception by making them feel uncomfortable in a comfortable environment, or vice versa [10]. This may result in the need for manual intervention using the SHC for comfort adjustment, thereby increasing electrical energy consumption and leading to loss of controller configuration, comfort, accuracy SHC, and other benefits offered by the utility. In addition, from the perspective of home office work discomfort can influence user/worker productivity. For these reasons, this article proposes the modeling of a comfort function that considers user perceptions of comfort through fuzzy logic.

The proposed comfort function is integrated in an SHC that uses PSO, as in [6,16], to schedule residential loads with the dual aims of savings and comfort. The analysis and validation of the proposed function includes different scenarios in terms of economy–comfort and load displacement, and considers energy tariffs, preferences, and user perceptions.

Thus, in this paper we present the following contributions to the state-of-the-art with respect to residential comfort analysis:

- Introduction of a novel residential comfort function capable of integrating parameters associated with the human perceptions of temperature and humidity.
- Proposal of a multi-objective SHC model that relies on PSO for scheduling the residential loads and integrates the proposed comfort function by means of fuzzy logic.
- Improvement of thermal comfort indices, the computational burden of the SHC, the efficiency of the PSO algorithm, and economic savings when compared with [5,6].
- Supplemental materials are available at GitHub^®^ through https://github.com/jonathacosta/SmartGrid/tree/main/SCC-SHC accessed on 15 February 2023, including our source codes, allowing prompt reproduction of our results.

The remainder of this paper is organized as follows. Section 2 presents a brief overview of the theoretical foundations. Section 3 presents the PSO and fuzzy principles along with mathematical definitions. Section 4 describes the methodology used to obtain the new comfort function and incorporate it into the SHC. Section 5 presents the results obtained under different scenarios. A discussion of the results is presented in Section 6 and our final considerations are provided in Section 7.

## 2. Demand-Side Management (DSM)

Among the main programs, DSM stands out for the response to tariff signals offered by the concessionaire to end users, namely, time of use (ToU), CPP, and RTP [17].

In the Brazilian market, the “white-rate tariff” is a term for the ToU [18] tariff, as mentioned above. In this scenario, low-voltage consumers, called group B, are informed of energy costs based on the day and time of consumption. Such a program provides significant benefits by shifting consumption from peak periods to times when the power distribution system has spare capacity.

Table 1 shows that as more loads are shifted to off-peak hours and the difference between the CT and ToU tariffs increases, the savings for consumers increase as well [18]. Otherwise, high energy costs may be incurred, which is why the use of SHC is of great importance in this scenario.

### 2.1. Smart Home Controllers (SHCs)

The basic definition of an SHC is based on the search for the best control strategy, considering the aim of scheduling household loads while minimizing cost and maximizing user comfort. In this sense, residential loads have a certain degree of flexibility in terms of duty cycle, power, and on/off times. These loads are part of the SH, which can exchange information with the utility in response to DSM programs. At this point, it should be emphasized that SH can include DG units with or without ESS and EV to support the optimization goal of SHC.

In this context, in [19] the authors proposed a simulation tool for residential load scheduling that uses BPSO and allows the proper selection of DG units. However, the mathematical model proposed in the tool does not have a function to evaluate the comfort level of the user. In [3], an event-based controller using PLI was proposed to minimize residential energy consumption while considering the variation in the electricity tariff and the average consumption of scheduled loads. However, the authors did not present user comfort metrics, and the proposed approach does not minimize consumption peaks, although it uses a restriction to avoid network overloads.

The authors of [20,21] employed a mixed PLI to optimize the operation of residential loads based on consumer preferences, which were modeled in a LSSW of HVAC equipment. The assessed scenarios combined DG, ESS, and EV. In [21], a thermodynamic comfort model for thermostatic loads was incorporated using PL, where the index PMV was used to measure the thermal comfort. However, this work did not consider users’ overall comfort level.

The authors of [22,23] proposed a dynamic parameter selection process to improve the performance of an HVAC controller designed for load balancing within one hour. However, comfort and consumption optimization functions were not included. In [24], MINLP is used while taking into account changes in the intraday energy price and user preferences for thermal comfort in order to minimize daily energy demand. The authors did not examine overall user comfort and did not consider perceived user comfort.

A linear aggregation function with weights in the range [0,1] was incorporated into the load controller by [4] to correlate the consumption and comfort functions; however, the authors did not address the perceived comfort of users. In [25], the authors introduced an equilibrium parameter and a comfort model using the Taguchi loss function. Their proposal relied on convex optimization, and they studied the rate scenarios DAP and FP. The authors did not investigate the users’ perceptions of comfort, and only considered pre-configured devices registered in the SHC. The controller evaluated in [26] used GA, TLBO and PL for load scheduling, and the authors proposed a novel optimization technique based on the combination of GA and TLBO called TLGO. The proposed technique presents a satisfaction cost index depending on the time and the type of device, where the user can use more electrical devices or use the same device for a longer period of time. The studies reported in [27] present a HIC based on the ubiquity concept to enable SH users to monitor residential loads and comfort metrics, as well as consumption and operational status related to fault alerts. The multi-objective controller described in [5] includes a global comfort function, while the work in [4] evaluated multiple fare scenarios associated with DG. The authors considered the user’s comfort level based on the relevance of each load to that comfort level. In these proposals, however, the relevance of loads was fixed and the user’s perceived comfort was not examined. The method introduced in [5] was reformulated in [28] by implementing an GA-based approach. This approach applies Pareto front approximation for the automatic definition of load activation, and provides users with options that take into account both energy consumption and residential comfort. According to [6], it is possible to associate PSO with the mathematical formulation of comfort and loads presented in [5], where it is possible to obtain a maximum consumption threshold modeled by an inverted Gaussian. Similarly, the authors did not address the users’ perception of comfort.

In [29], the authors presented a residential controller that optimizes the charge/discharge scheduling of local ESS in SH with DG. It aims to it aims to minimize the cost of local energy consumption and to enable the negotiation of excess energy between prosumers and between prosumers and the utility. The authors used an objective function based on MOGWO and compared the results with PSO. The authors did not, however, examine users’ perceptions of comfort

A coordination model involving a central controller, local controllers, and residential consumers was proposed in [30]. The hierarchical structure aims to reduce demand through competitive bidding. The authors modeled the consumption of air conditioners and water heaters using an optimization function based on MILP to minimize the total reward at each bid.

Lyapunov optimization based on virtual queue stability was used in [31]. This work aimed to study energy optimization in an SH with HVAC loads, DG, and an ESS. The proposed algorithm creates and controls four queues for indoor temperature, EV charging, and ESS. System implementation is conducted by convex programming. The authors of [32] employed fuzzy logic to determine the estimated comfort and cost–benefit ratio in the near future, and used SA to determine the set point for battery bank operation. Their focus was on maximizing the monthly profit from energy sales. In turn, the study of [33] presented an algorithm that reduces the consumption of HVAC loads. The resulting energy surplus enables the adjustment of the illuminance set point in the range of 0–500 lux to maximize the user’s visual comfort. Both of the aforementioned controls are based on fuzzy logic. Finally, in [34], a controller was proposed that considers DG and ESS to minimize the energy cost. The load schedule results from the proposed algorithm based on ACO and TLBO. The authors studied comfort in terms of thermal and lighting preferences, though they did not consider the user’s perception of comfort.

### 2.2. Comfort Analysis Strategy

Based on the above, residential comfort can be classified into three categories: (a) Conceptual Comfort, which refers to the wellbeing of the user and the perception of less effort. An example is communication and automatic configuration between IoT devices in a SH that does not require specific knowledge of the user, as in [27]. This type of convenience, by its nature, does not include quantitative metrics. (b) Individual Comfort, which refers to the individual measurement of variables associated with user comfort, that is, temperature, humidity, lighting, noise, and CO${}_{2}$ levels, among others. Each variable can be analyzed under a specific metric, as in [31,33]. (c) Global Comfort, which refers to a numerical index representing the sum of the individual values associated with each residential load enabled according to user preferences, as in [5,6,25]. In this category, each residential load ($x_{i}$) is assigned a value representing its relevance level ($w_{i}$), which can be expressed as the sum of the products $x_{i}\times w_{i}$.

Therefore, in the above research, it can be seen that while SHC algorithms and current strategies consider user preferences in comfort modeling, they do not consider user perceptions. The user’s comfort preferences have been considered when registering household loads in the SHC. On the other hand, the user’s comfort perception varies throughout the day and has not been considered in SHC modeling in the aforementioned studies. It is worth noting that the present work focuses on global comfort and aims at optimizing residential comfort by incorporating user-perceived variables, as presented in Section 1.

## 3. Computer Algorithms

The strategy presented in this paper to minimize energy costs and maximize user comfort consists of a combination of fuzzy logic and PSO. Below, we present the mathematical bases of PSO and fuzzy logic.

### 3.1. Principles of Fuzzy Sets and Logic

In classical (binary) logic, sets of elements are called crisp sets, in which it is defined that an element *x* belongs to a set *A* and not to a set *B*, as there is a well-defined boundary to decide whether or not element *x* belongs to a set, expressed in the following form: x∈A and x∉B. In fuzzy logic, sets of elements are called fuzzy sets, in which an element *x* has degrees of membership in set *A* and in set *B*, as the above boundary decision is not present. The degree of membership of an element *x* in a set *A* is expressed by $\mu_{A}\left(x\right)$, which takes continuous values in the closed interval [0,1]. Thus, an element *x* can be described by its degrees of membership in the sets *A* and *B*, which can be expressed, for example, in the form $\mu_{A}\left(x\right)\;=\;0.4$ and $\mu_{B}\left(x\right)\;=\;0.6$.

Furthermore, a linguistic variable is defined as a variable with values that are fuzzy set names (*U*), as in: *U* = {“cold”, “warm”, “very warm”}. Such variables refer to the diffuse human perception of measurable quantities, and may contain sets of logical connectives (negation: no, connectives: and/or), modifiers (much, little), and delimiters (such as parentheses). Otherwise, a function that establishes a relationship between crisp values and linguistic variables is defined as a membership function ($u_{A}\left(x\right)$) in fuzzy logic. This function expresses a priori knowledge about the behavior of the analyzed variable, and can be modeled by a profile: triangular, trapezoidal, Gaussian, generalized bell, sigmoidal, and others.

That said, mapping an input with a precise value (not fuzzy through membership functions ($u_{A}\left(x\right)$) is called the fuzzy step of a fuzzy inference system. This step processes rules in the linguistic variables to derive the output values, for which it uses the Mamdani or Takagi–Sugeno method. The first method returns a fuzzy set, while the second method returns a real number. The last step of a fuzzy inference system is called defuzzification, and can use one of several well-known methods for it: centroid, first-of-maximum, maximum criterion, angle bisector, and others.

#### 3.1.1. Formalization

Formally, a fuzzy set *A* in *X* is expressed as a set of ordered pairs, as shown in Equation (1):

(1) $$A=\{x,u_{A}\left(x\right)\;with\;x\in X\}$$

where $u_{A}\left(x\right)$ is the membership function of element *x* in the set *A*, which can be represented as in Equation (2) (triangular format), Equation (3) (trapezoidal format), Equation (4) (Gaussian format), and Equation (5) (generalized bell format):

(2) $$trimf\left(x;a,b,c\right)=max\left(min\left(\frac{x-a}{b-a},\frac{c-x}{c-b}\right),0\right)$$

(3) $$trapmf\left(x;a,b,c,d\right)=max\left(min\left(\frac{x-a}{b-a},1,\frac{d-x}{d-c}\right),0\right)$$

(4) $$gaussmf\left(x;a,b,c\right)=e^{-\frac{1}{2}{\left(\frac{x-c}{\sigma }\right)}^{2}}$$

(5) $$gbellmf\left(x;a,b,c\right)=\frac{1}{1+|\frac{x-c}{b}{|}^{2b}}$$

where: a,b,c,d represent the limits of functions on the Cartesian axis.

#### 3.1.2. Application

**Figure 1:**
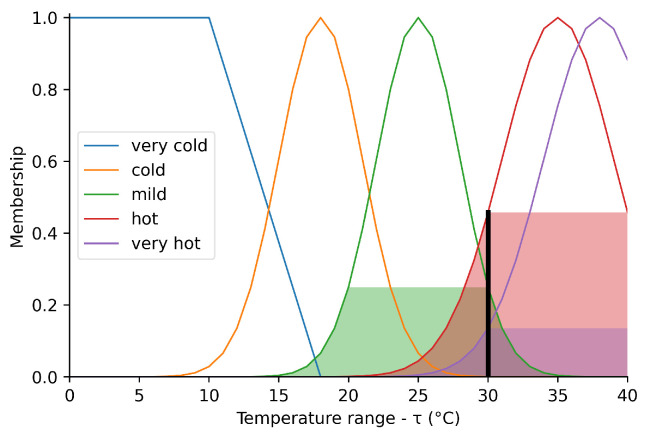
Perceived temperature.

Fuzzy inference systems are commonly used in many areas, for example, in the development of control systems for air conditioners, washing machines and vehicles. In the context of this paper, Figure 1 illustrates human perception of temperature in a residential environment. The fuzzy degrees of membership in the “cold” and “warm” groups are represented by the hatching in the Gaussian lines in the center of the figure.

Note that the values corresponding to a temperature of ≈30 °C (a value of crisp on the ordinate axis) have a membership degree of $\mu_{mild}\left(temp\right)\approx 0.26$, $\mu_{hot}\left(temp\right)\approx 0.49$, and $\mu_{very\_hot}\left(temp\right)\approx 0.15$ on the vertical axis (‘membership degree’).

Direct application of LF and a link to the implemented source codes are provided for those involved in related research. In addition, modeling and control of systems with fuzzy logic are available in [35,36], and introductory concepts and a methodology for applying fuzzy logic in the context of DSM are presented in [37,38,39].

### 3.2. Principles of Particle Swarm Optimization (PSO)

PSO is a stochastic population optimization method based on the collective behavior of animals, such as schooling or flocking [40]. The algorithm attempts to find the best solution using a population of particles, and is based on the concept of collective cooperation.

#### 3.2.1. Formalization

In the PSO algorithm, a particle *i* is defined by the vectors of position ($\vec{x_{i}}$), velocity ($\vec{v_{i}}$), and best individual position ($\vec{pbest}$). A vector with the best global position of the swarm ($\vec{gbest}$) is the result of the individual positions of *n* particles in this cluster. The elements of the algorithm are:

A         The population of particles
$\vec{x_{i}}$         The position vector of particle *i* in the solution space
*f*          The evaluation function (fitness)
$\vec{v_{i}}$         The velocity vector of particle *i*
$\vec{pbest_{i}}$  The vector of the best individual position of particle *i*, corresponding to the position in the search space where particle *i* has the best value of the evaluation function *f*.
$\vec{gbest_{i}}$  The vector of the best global position of the particle, corresponding to the position that provides the best value among all $pbest_{i}$.

#### 3.2.2. Updating Individual and Global Best Positions

Equations (6) and (7) specify how $pbest_{i}$ and $gbest_{i}$ are updated with time *t*. The swarm is said to have *n* particles in a minimization problem of a function *f* such that:

(6) $$\begin{array}{c} \vec{pbest_{i}}\left(t+1\right)=\left\{\begin{array}{cc} \vec{pbest_{i}}\left(t\right) & \mathrm{if}\;f\left(\vec{pbest_{i}}\left(t\right)\right)\leq f(\vec{x_{i}}\left(t+1\right))\\ \vec{x_{i}}\left(t+1\right) & \mathrm{if}\;f\left(\vec{pbest_{i}}\left(t\right)\right)>f\left(\vec{x_{i}}\left(t+1\right)\right) \end{array}\right. \end{array},$$

(7) $$\begin{array}{c} \vec{gbest_{i}}\left(t+1\right)=min\left\{f\left(\vec{pbest}\right),f\left(\vec{gbest}\right)\right\} \end{array},$$

where $\vec{pbest}\in \left\{\vec{pbest_{0}},\;\vec{pbest_{1}},\;\cdots ,\;\vec{pbest_{n}}\right\}.$

#### 3.2.3. Updating the Velocity and Position of a Particle

In addition, the velocity ($\vec{v}$) for a particle *i* is defined by the inertia parameter, the cognitive parameter, and the social parameter. The first (inertia parameter) is the previous velocity of the swarm, which causes the particle to continue to move in the same direction as it already is. The second (cognitive parameter) expresses the particle’s individual experience of where the solution lies, and influences the particle to move to a better position than its current one. The third parameter (social parameter) represents the experience of the swarm, and influences the particle to follow in the direction of its best neighbors. Equations (8) and (9) respectively describe how the velocity and position of the *i*th particle are updated:

(8) $$\begin{array}{c} \vec{v_{i}}\left(t+1\right)=w\vec{v_{i}}\left(t\right)+c_{1}r_{1}\left(\vec{pbest_{i}}-\vec{x_{i}}\right)+c_{2}r_{2}\left(\vec{gbest_{i}}-\vec{x_{i}}\right) \end{array}$$

(9) $$\begin{array}{c} \vec{x_{i}}\left(t+1\right)=\vec{x_{i}}\left(t\right)+\vec{v_{i}}\left(t+1\right) \end{array}$$

where $c_{1}$ and $c_{2}$ are positive constants representing the cognitive and social parameters, respectively, the variables $r_{1}$ and $r_{2}$ are random numbers ∈[0,1], and *w* is the inertia weight.

#### 3.2.4. Application

General aspects regarding the development of the PSO algorithm, along with discussion of the constraint factors, inertia weights, dynamic tracking systems, adaptive parameter adjustments, and more, are available in [41,42]. In addition, [16,43,44,45] provide examples of the use of PSO and fuzzy strategies in other applications.

## 4. Proposed Method: Holistic Architecture

**Figure 2:**
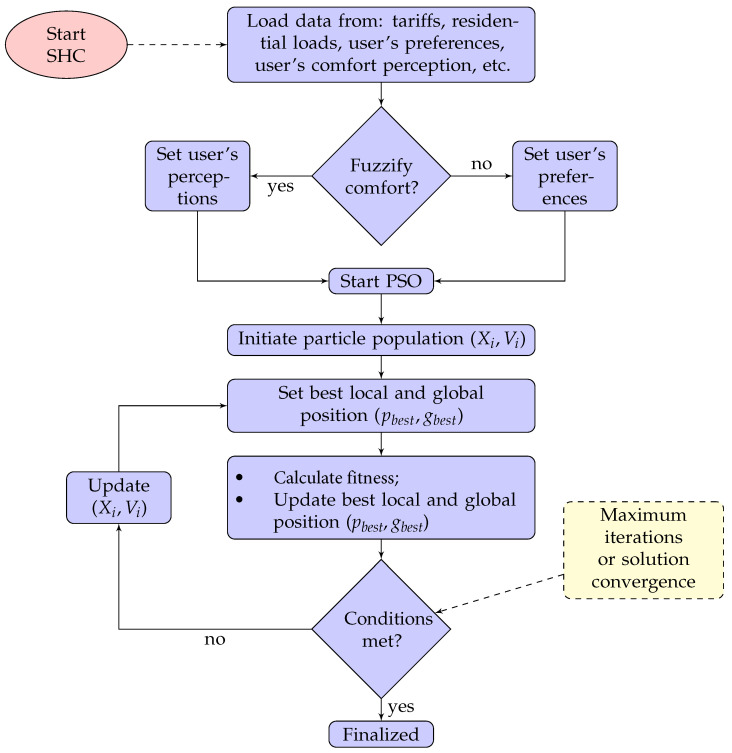
Flowchart of the combined PSO and fuzzy algorithm.

Figure 2 summarizes the results shown up to this point, and shows the user interaction of the SHC model.

Note the first decision block of the flowchart; after loading the user’s preference and perception values, PSO can achieve a tradeoff between savings and comfort using the comfort conventional concept according to user preferences or the new comfort concept according to user perception.

### 4.1. SHC Structure Diagram

**Figure 3:**
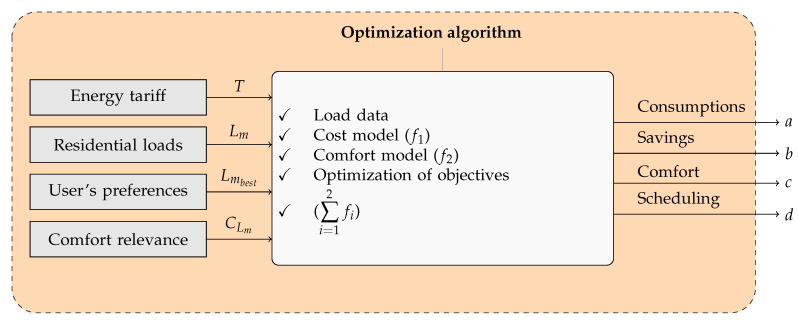
Proposed SHC model.

Figure 3 provides an overview of the model proposed in this paper.

The SHC receives information about utility billing (*T*), residential loads ($L_{m}$), residential load activation preferences ($L_{m_{best}}$), and comfort level ($C_{L_{m}}$). The residential loads considered here ($L_{m}$) include: (a) schedulable loads that can be directly activated by smart outlets at a specified time interval, such as air conditioners, heaters, and pool filter pumps, among others; and (b) non-schedulable loads that cannot be directly controlled, such as multimedia equipment, microwave ovens, toasters, vacuum cleaners, etc. The PSO algorithm is used for multi-objective optimization of schedulable loads. For this purpose, the data comprising the residential loads, consumption profile ($f_{1}$), and residential comfort profile ($f_{2}$) are modeled, leading to an appreciation of consumption (*a*), savings (*b*), and comfort (*c*) by a day-ahead load schedule (*d*) considering both asynchronous operation and synchronous operation throughout the same day.

### 4.2. Mathematical Modeling

The mathematical model of the SHC is represented as a discrete-time system operating at a sampling rate $T_{s}$ that allows the management of controllable and non-controllable loads according to the residential consumption profile. In the proposed approach, the cost function ($f_{1}$) and the comfort function ($f_{2}$) define the financial savings and the comfort relevance level, respectively, according to Figure 3. Table 2 defines the variables used in the modeling.

#### 4.2.1. Cost Model—f${}_{1}$

This paper uses the mathematical definitions of residential load at the grid level, as presented in [3]. The mathematical model of residential loads corresponds to Equation (10), considering the following premises: *m* schedulable loads, *N* daily samples, and a sampling rate $T_{s}$, as well as the notation described in Table 2, as follows:

(10) $$f_{Fcost}=\sum_{m=1}^{M}\sum_{k=I_{Cm}}^{I_{Cm}+N_{m}}\left({\bar{P}}_{m}\left[k\right]\frac{T_{s}}{60}C\left[k\right]\right)$$

subject to the following constraints:

(11) $$I_{Sm}\leq I_{Cm}\leq I_{Em}$$

(12) $$\sum_{k=1}^{N}\left(\sum_{m=1}^{M}{\bar{P}}_{m}\left[k\right]\right)\leq P_{k}$$

The constraints in Equation (11) state that the schedule for activation of the *m*-th load must be within the minimum and maximum flexibility intervals defined by the user, while the loads must not exceed the threshold demand at the *k*-th time of activation according to the constraints of Equation (12).

The cost function ($f_{1}$) defines the economic savings due to SHC. The first and second terms in Equation (13) correspond to the costs resulting from the user preference profile and the SHC scheduling, respectively:

(13) $$f_{1}=\sum_{m=1}^{M}\left(\sum_{i=I_{Bm}}^{I_{Bm}+N_{m}}\left({\bar{P}}_{m}\left[i\right]\frac{T_{s}}{60}C\left[i\right]\right)-\sum_{i=I_{Cm}}^{I_{Cm}+N_{m}}\left({\bar{P}}_{m}\left[i\right]\frac{T_{s}}{60}C\left[i\right]\right)\right)$$

where $f_{1}\geq 0$, meaning that the schedule proposed by the SHC is accepted by the algorithm as a valid solution for the user.

#### 4.2.2. Comfort Model—f${}_{2}$

The comfort model proposed in [5] considers the comfort relevance level of a load *m* as a fixed value and defines user preferences. It takes into account the user’s perception of comfort, for example, in terms of temperature and humidity. Such parameters may change the relevance that the user assigns to a given load *m* when registering it through the SHC. Considering the above, the second objective function ($f_{2}$) defines the relevance of comfort ($C_{Lm}$) using fuzzy logic.

For this purpose, the user should register the residential loads that can be scheduled in SHC, as well as the comfort relevance values ($0\leq C_{Lm}\leq 1$) and the load onset times in terms of minimum ($I_{Sm}$), maximum ($I_{Em}$), and preferred ($I_{Bm}$) values. The $C_{Lm}$ value of each load is updated using fuzzy logic taking into account the user’s perception of comfort.

Equation (14) represents the comfort function. The first term corresponds to the activation window of a load *m* with respect to the user’s preferences; thus, this value is used as a reference for calculating the comfort. The second term defines the distance between the time instant ($I_{Cm}$) selected by SHC and the time preferred by the user ($I_{Bm}$), which is weighted by the comfort relevance of the *m*-th load.

(14) $$f_{2}=\left[max(\left|I_{Sm}-I_{Bm}\right|,\left|I_{Em}-I_{Bm}\right|)\right]-C_{Lm}\left|I_{Cm}-I_{Bm}\right|$$

For a given load *m* with a comfort relevance of $C_{Lm}=1$, this parameter takes the maximum value when the SHC’s scheduled time converges with the user’s preferred time ($I_{Cm}\approx I_{Bm}$). Otherwise, if $I_{Cm}\approx I_{Sm}$ (or $I_{Cm}\approx I_{Em}$ at the other end of the load activation window), the comfort is minimal, as the operation cycle starts at the time farthest from the one the user has specified as their preferred time.

#### 4.2.3. Fuzzification of Comfort Relevance Level

**Figure 4:**
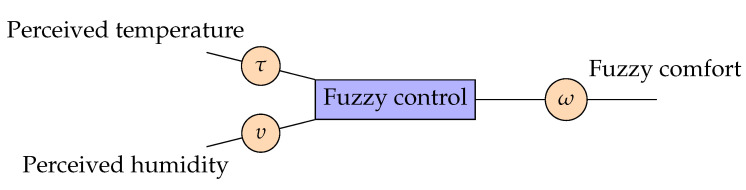
Fuzzification of the comfort relevance level.

Figure 4 represents the fuzzification process of the comfort relevance level ($C_{Lm}$) considering user perception.

The values denoting the user’s perception of temperature and humidity are the inputs of the fuzzy system by means of the linguistic variables τ and υ, respectively. The fuzzy system uses rules to calculate a new relevance value (ω) for a load *m*, then updates the value of $C_{Lm}$ from Equation (14).

The values of τ and υ can be loaded automatically or passed to the SHC by the user. In the first case, the proposed algorithm accesses the weather data available at https://pt.weatherspark.com/ and https://tempo.inmet.gov.br/TabelaEstacoes/82397, accessed on 15 February 2023, to load the temperature and humidity values associated with τ and υ, respectively. In the second case, SHC interacts with the user through voice commands to collect information about the user’s temperature and humidity perceptions, then assigns values to the respective variables. The user’s perceptions are fuzzy variables that can be handled with the linguistic variables defined in Table 3. When the user starts interacting with the SHC, a timer is activated to ensure that the first mode of operation is loaded in the event that the process is not completed because the user gives up or times out. The comfort relevance stage update phase (ω) is the first stage to be executed by the SHC algorithm. The update from $C_{Lm}$ to ω is performed according to the criterion defined by Equation (15), meaning that only the registered loads with priorities close to the maximum relevance value ($C_{Lm}=1.0$) are activated.

(15) $$C_{Lm}=\omega ,\;\forall \;C_{Lm_{0}}\geq 0.5$$

where $C_{Lm_{0}}$ is the initial value of the relevance comfort level of the *m*-th load registered by the user and ω is the value of the relevance comfort level comprising the fuzzy sets of the user’s perception.

### 4.3. Comfort Fuzzification Model

The values of $C_{Lm}$ obtained from Equation (14) are updated considering fuzzy sets for the perception of thermal comfort (τ) and humidity (υ), the linguistic variables of which are defined by the corresponding notation and normalized range shown in Table 3.

The linguistic variables of the fuzzy sets were quantitatively distributed using the intuitive method to investigate different combinations between them while considering the following definitions: $\tau =\{t_{1},t_{2},t_{3},t_{4},t_{5}\}$, $\upsilon =\{\upsilon_{1},\upsilon_{2},\upsilon_{3}\}$. The ranges of linguistic variables for each fuzzy set (τ,υ,ω) were determined using Gaussian, trapezoidal, and triangular membership functions such that the thresholds converge with those defined by the corresponding standards of the ABNT (NBR 16401-2/2008) and the ASHRAE [46]. The adjustment of the membership functions and the choice of generators for each membership function were accomplished by considering successive simulations using the exhaustive search method for each function and domain.

The fuzzy rules were created both by directly combining the two input variables and combinations between them. Thus, comfort relevance, that is, the output variable ω, always takes into account the influence of the input variables. Next, we incorporate the following rules.

1. If $\tau \leq t_{2}$ and ∀υ, or if $\tau =t_{3}$ and $\upsilon =u_{3}$, then $\omega =c_{1}$
2. If $t_{3}\leq \tau \leq t_{4}$ and $u_{1}\leq \upsilon \leq u_{2}$, then $\omega =c_{2}$
3. If $\tau =t_{5}$ and ∀υ, or if $\tau =t_{4}$ and $\upsilon =u_{3}$, then $\omega =c_{3}$

It can be observed that the output of the “low” ($c_{1}$) comfort level (ω) in (1) is when the user considers the ambient temperature as “cold” or “very cold” ($\tau \leq t_{2}$ ), regardless of the perceived humidity (ϵ). In this way, the rule establishes a relationship between user-perceived temperature and humidity, meaning that it provides the SHC with the flexibility to schedule loads by reducing them (ω). The remaining rules follow the same reasoning for creating the inference system.

**Figure 5:**
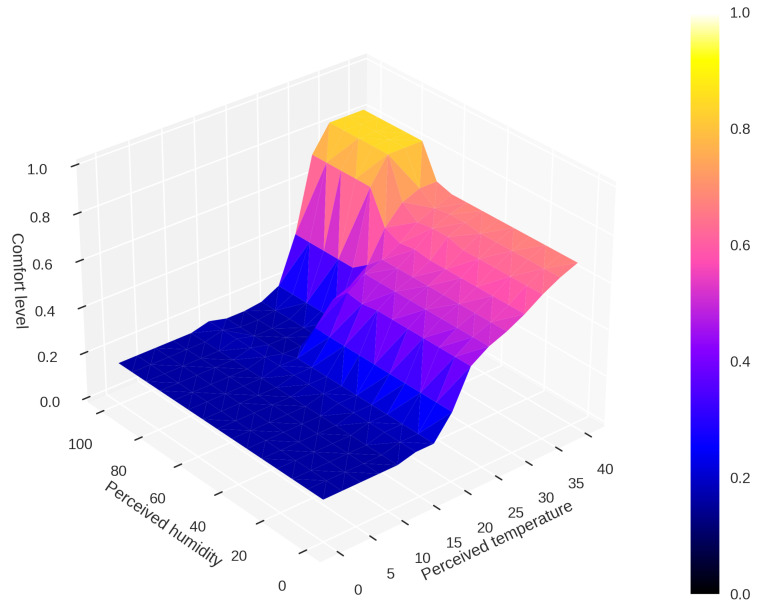
Fuzzy control surface as a function of user perception.

Figure 5 shows a surface map based on the relationship between user perception and the comfort adjustments made in the SHC.

The user’s comfort level is determined by defuzzification of the temperature and humidity variables perceived by the user. Following the fuzzification rules presented in this section, the surface map in Figure 5 is obtained. It is worth noting that the axes represent temperature, humidity, and comfort. In addition, user perception allows the comfort level to be adjusted even in fuzzy modeling, providing the SHC with the flexibility to reduce energy consumption in residential energy usage contexts.

**Figure 6:**
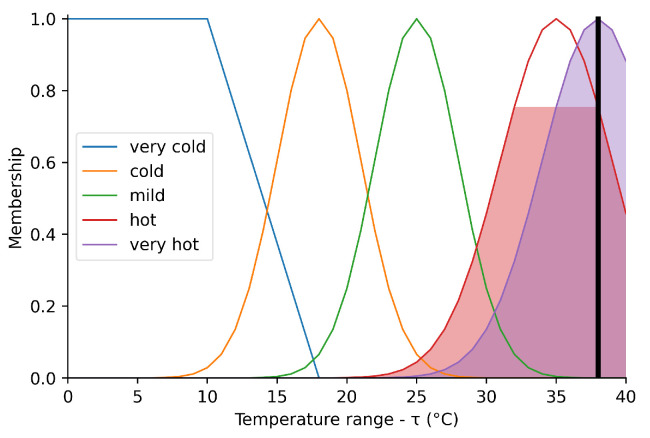
Perceived temperature.

Figure 6 shows the behavior of the fuzzy set representing the temperature. The domain corresponds to the range between 0 and 40 °C, while the membership function is between 0.0 and 1.0.

The fuzzy input ‘very cold’ is modeled by a trapezoidal function starting at 0 °C and decreasing from 10 to 18 °C. The fuzzy input ‘cold’ is modeled by a Gaussian function centered at 18 °C with a deviation of 3 °C. The fuzzy input ‘mild’ is modeled by a Gaussian function centered at 25 °C and deviating by 3 °C. The fuzzy input ‘hot’ is modeled by a Gaussian function centered at 35 °C and differing by 4 °C. The fuzzy input ‘very hot’ is modeled by a Gaussian function centered at 38 °C and differing by 4 °C. The shaded areas represent the sets of membership corresponding to the crisp input of 38 °C. This input is represented by the vertical bar on the abscissa axis, for which the projection on the ordinate axis represents its membership in the sets that contain it, that is, $\mu_{hot}\left(\tau \right)\approx 0.75$ and $\mu_{veryhot}\left(\tau \right)\approx 1.0$.

**Figure 7:**
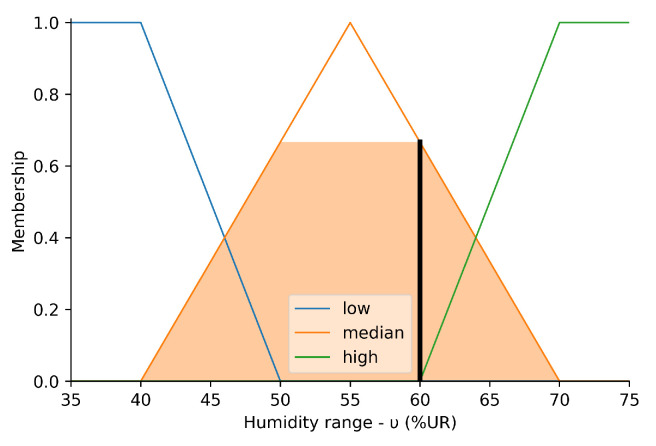
Perceived humidity.

Figure 7 shows the behavior of the fuzzy set named ‘humidity’. The domain corresponds to the range between 0 and 100% of the RH, while the amplitude includes the membership values between 0 and 1.

The ‘low’ input is modeled by a trapezoidal function that starts at 0 and decreases between 40 and 50% RH. The ‘medium’ input is modeled by a triangular function centered at 55% RH and bounded between 40 and 70% RH. The ‘high’ input is modeled by a trapezoidal function that starts at 60, increases to 70% RH, and remains constant at 75% RH. The shaded area represents the membership set defining the crisp input of 60% RH. This input corresponds to the vertical bar on the abscissa axis, and its projection on the ordinate axis represents the membership of the sets containing it, that is, $\mu_{medium}\left(\upsilon \right)\approx 0.65$.

**Figure 8:**
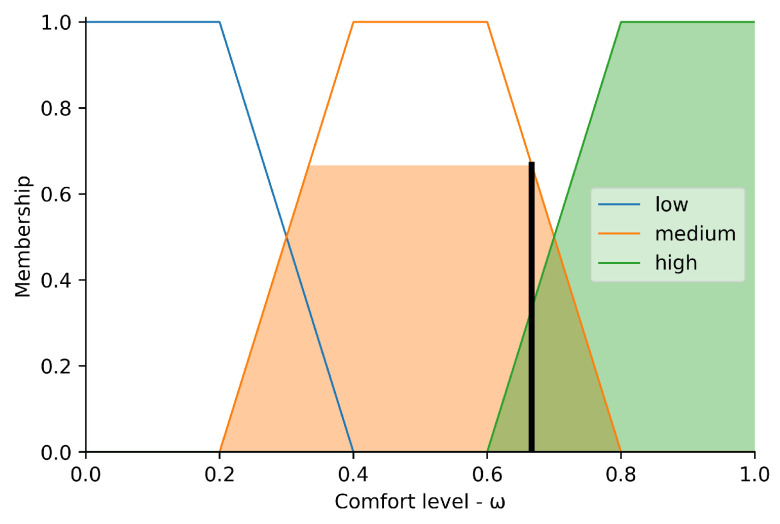
Comfort level.

Figure 8 displays the behavior of the fuzzy set called ‘comfort’. Both the domains and the amplitude range between 0.0 and 1.0.

The ‘low’ input is modeled by a trapezoidal function that starts at 0 and decreases between 0.2 and 0.4. The ’medium’ input is modeled by a trapezoidal function that starts at 0.2, increases to 0.4, remains constant at 0.6, and decreases to 0.8. The ‘high’ input is modeled by a trapezoidal function that starts at 0.6, increases to 0.8, and remains constant at 1.0. Unlike the previous graphs, the shaded area represents the membership sets corresponding to the crisp output of 0.83. This value is represented by the vertical bar on the abscissa axis, and its projection on the ordinate axis represents the membership of the sets containing it, that is, $\mu_{comfort}\left(\omega \right)=1.0$.

The proposed fuzzy modeling relies on an OOP approach. The source code was written in the Python^®^ language using the Scikit-Fuzzy, numpy, and random modules. The developed classes and methods are appropriately commented in the code for proper understanding of the programming logic, which includes a simulation field for new values of the fuzzy variables. The source code is available at https://raw.githubusercontent.com/jonathacosta/SmartGrid/main/SCC-SHC/Codes/ModConfFz.py, accessed on 15 February 2023.

The following definitions identify each of the scenarios assessed in the simulations described in Section 5:

- fz-comf: employs fuzzy variables to assign a new value to the comfort relevance level according to Equation (15)—user’s perceptions.
- nfz-comf: a fixed value for the comfort relevance level is used, which is specified by the user when registering the loads in the SHC—user’s preferences.
- PSO & nfz-comf: the algorithm proposed in [6], which relies on PSO and the comfort function proposed in [5], which uses nfz-comf.
- PSO & fz-comf: the algorithm introduced in this paper, combining PSO and the comfort function proposed.
- PSO & nfz-tag-comf: the algorithm proposed in [6], which relies on PSO and the comfort function proposed in [25], which uses nfz-comf.
- PSO & fz-tag-comf: the algorithm presented in this paper combining PSO and the comfort function proposed in [25], with the addition of fuzzy comfort (fz-comf).

### 4.4. Multi-Objective SHC Function

The evaluation function of the solutions obtained with the SHC consists of the functions $f_{1}$ and $f_{2}$. The first defines the resulting financial savings, while the second represents the user’s comfort level. Equation (16) represents a total cost function *F* that consolidates $f_{1}$ and $f_{2}$, corresponding respectively to Equations (13) and (14).

(16) F=α·f1+(α−1)·f2

where α∈[0,1]. The values adopted for α are directly related to the relationship between consumption and user comfort. Comfort is maximal for the maximum threshold α=0.0, resulting in minimal or no savings. Conversely, comfort is minimal for α=1.0, while savings are maximal.

The pseudocode of SHC which applies the proposed comfort function is shown in Algorithm 1.
**Algorithm 1:** Pseudocode of SHC based on fuzzy logic  **  Input**   :Loads, Tariff(T), Population(P), Iterations(It), c1, c2    **Output**:  gBest **1**  **begin** **2**         **for**
i=1to(Loads)
**do** **3**               **if**
*$C_{lm}>0.5$*
**then** **4**                    $C_{lm}\leftarrow \omega$; **5**         pop ← iniPop(Loads) **6**         fitness ← calcFitness() **7**         Determine pbest and gbest **8**         k←0 **9**         output←0 /*Convergence*/ **10**        **while**
*k<=It & output = 0*
**do** **11**               w = diw_InertialTechnique() **12**               **for**
i=1toP
**do** **13**                         (**if**
*$fitness_{i}>gBest$*
**then** **14**                                 ($gbest\leftarrow pop_{i}$; **15**                                 last←k **16**                         **if**
*$fitness_{i}>pBest$*
**then** **17**                                 ($pbest\leftarrow pop_{i}$; **18**                         r1,
*r*2←rand() **19**                         Update $pop_{i}\cdot V$ and $pop_{i}\cdot X$ **20**                         fitness←calcFitness() **21**                         **if**
*(k−last)>(0.1×It)*
**then** **22**                                 **if**
*k<=(0.2×It)*
**then** **23**                                         /* Restart P keeping the current gbest as the worst solution */                                            
pop←iniPop(Loads); **24**                                         fitness←calcFitness(); **25**                                         p←argmin(fitness); **26**                                         $pop_{p}\leftarrow gbest$ **27**                                 **else** **28**                                         (output←1 /*Convergence*/ **29**                   
k←k+1

To ensure performance, note that the convergence criterion is 20% of the maximum iterations, with the population restarted if no improvement in the solution is achieved after 10% of the iterations.

## 5. Results

### 5.1. Simulation Scenarios and Analysis Criteria

The characteristics common to all simulation scenarios are: (a) sampling rate ($T_{s}$) 5.0 min; (b) daily demand threshold 4.0 kW, represented by an inverted Gaussian centered at 18:30 h with an amplitude of 1.0 kW to simulate a decrease in the demand threshold; (c) the ToU tariffs; and (d) the household loads described in Table 4 and detailed in [5].

#### 5.1.1. Simulation Parameters

Reference loads are identified by the operating cycle, time per cycle (Δt) in minutes, average power ($\bar{P}$) in kW, maximum power ($\hat{P}$) in kW, best start time, minimum time, maximum time, and comfort relevance level ($C_{Lm}$). In addition, certain loads have two values for the best start time. Our simulations were performed with the same parameters presented in [6] to allow for a more fair comparison, considering the following aspects: (a) swarm size of ten particles, with each particle representing one possible solution of the algorithm, i.e., the total number of loads; (b) the weight change technique (*w*) and inertial weight (DIW); (c) the cognitive and social parameters ($c_{1}$ = 2 and $c_{2}$ = 2, respectively); (d) a maximum number of iterations of 10,000. Each solution in each scenario was executed 30 times.

#### 5.1.2. Analysis Criteria

Each solution was evaluated based on the results of the simulated scenarios. The same simulation parameters were used for each scenario, with a single variable being changed each time to allow for fair comparison of the results. For this purpose, the fitness function, financial savings, relative comfort, expected consumption, total consumption of household loads, execution time, and standard deviation were analyzed for each solution. The scenarios were evaluated against the above metrics using load scheduling diagrams and exploratory data analysis according to the model developed by the authors. The corresponding source code is available at https://raw.githubusercontent.com/jonathacosta/SmartGrid/main/SCC-SHC/Codes/ModEDA.py, accessed on 15 February 2023.

### 5.2. Analysis of Comfort Relevance Level

From Table 4, it is apparent that air conditioners have a relevant comfort level of ($C_{Lm}$) 1.0. This means that the SHC must prioritize the activation of these loads at the time chosen by the user in order to satisfy the user’s comfort preferences. However, the user’s comfort perception may vary over the course of days due to both climatic and psychological aspects. This means that the previously established comfort preferences may not match the individual’s actual comfort perception at a given time. For example, the relevance of air conditioning must be adjusted to the user’s comfort perception. Table 5 shows the variation in the relevance of user comfort level ($C_{Lm}$) for an air conditioner along with the fuzzy variables involved.

It can be observed that the temperature (τ) and humidity (υ) perceptions were chosen under extreme conditions of the domain ranges. In addition, all loads are scheduled to ensure comfort. However, because $C_{Lm}$ is assigned with the value of ω, the load scheduling algorithm has the flexibility to shift loads to times close to peak hours, maximizing user comfort while simultaneously allowing for improvement of the cost–comfort ratio.

### 5.3. Analysis of Residential Scenarios

The simulations in this scenario consider the most critical tariff costs published by a regulatory agency in northeastern Brazil, namely, USD 0.3047 for green flag tariffs, USD 0.3073 for yellow flag tariffs, and USD 0.3230 for red flag tariffs [47].

It is useful to note that the use of the white tariff (ToU) allows an additional adjustment of the cost–comfort ratio by α according to Equation (16). This parameter was used in this simulation as [0.25,0.50,0.75] to highlight different SHC configuration conditions between two houses with the same loads and the same fuzzy parameters. With the parameter α=0.25, according to Equation (16), the user sets SHC to 0.25 for cost savings and 0.75 for comfort. Therefore, the user configures the system to prioritize user comfort over savings in load scheduling. The reverse is true for α=0.75, and for α=0.5 there is an even threshold.

#### 5.3.1. ToU Scenario com α=0.25

With the parameter α=0.25, the user sets the SHC to 0.25 for cost savings and 0.75 for comfort. This configures the system to favor comfort over cost savings when scheduling loads. Table 6 shows the results of the simulations with the above parameters.

Table 6 shows the values and compares two different comfort functions, the first originally proposed by [5] and the second proposed by [25]. Note that the average user’s comfort level is higher when the SHC uses the fz-comf comfort function instead of the nfz-comf comfort function. Even when the ω parameter tends to 0.0, the user continues to gain comfort. The same relationship is observed when the SHC sets fz-tag-comf instead of nfz-tag-comf. In this scenario, it can be seen that the use of the proposed comfort function provides further benefits, as it takes into account the user’s perception of comfort in load scheduling instead only the user’s preferences.

#### 5.3.2. ToU Scenario com α=0.50

Otherwise, using the parameter α=0.50, the user sets the SHC to 0.50 for cost savings and 0.50 for comfort. In this way, the system can be configured to balance cost savings and comfort in load scheduling. Table 7 shows the result of the simulations for the above parameters.

It can be observed that the use of the proposed comfort function in this scenario is more beneficial when the SHC operates with the fz-tag-comf function, as it takes into account the user’s comfort perception, increases the average comfort, and reduces the load scheduling cost.

#### 5.3.3. ToU Scenario com α=0.75

Otherwise, with the parameter α=0.75, the user sets the SHC to 0.75 for cost savings and 0.25 for comfort. This configures the system to prioritize cost savings over comfort when scheduling loads. Table 8 shows the results of the simulations with the above parameters.

Thus, in this scenario where cost savings are the main concern, it is advantageous for the SHC to use only the nfz-tag-comf comfort function, as it provides the greatest savings at the highest comfort level. This means that the SHC does not take into account the user’s perception of comfort, only their preferences as predefined in the load registration, guaranteeing an average comfort level while focusing on reducing the cost when scheduling loads.

## 6. Discussion

Simulations were performed considering all possible combinations between ToU energy tariffs, user preferences in terms of cost–comfort, user perception, and number of loads in a residence. The results confirm our initial hypothesis that the use of a comfort function integrating the above variables allows user comfort to be maximized without affecting cost.

**Figure 9:**
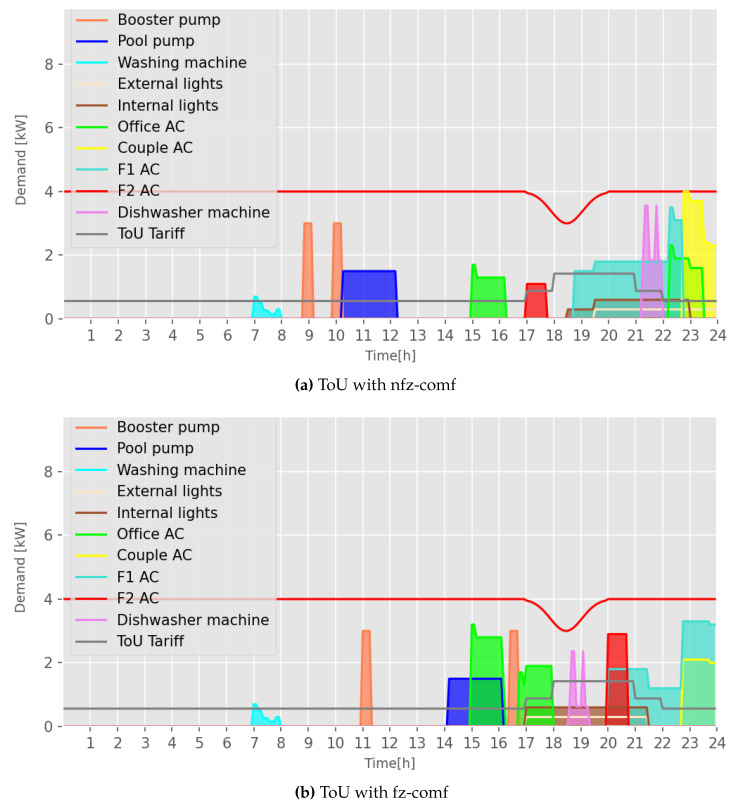
Load scheduling performed by the SHC using the ToU tariff and α=0.25.

In addition, improved performance leads to more flexible load scheduling, which allows shifting loads to times closer to user preferences. Figure 9a,b shows the load scheduling graphs for scenarios with and without fuzzy comfort considering the tariff ToU, α=0.25, and 10 loads. The range in the graphs between 17:00 (5 PM) and 22:00 (10 PM) corresponds to the highest tariff costs. Therefore, SHC should reduce energy consumption during critical periods and/or shift loads to off-peak hours.

The red area in Figure 9a,b represents an air conditioner (‘F2 AC’) that should be put into operation at 17:00 (see Figure 9a). The application of fz-comf allows the load to be shifted to near 20:00, where 20:00 is the user’s preferred time according to Table 4 (Id 7), maximizing thermal comfort. This process occurs at the algorithm level; a comfort relevance level mitigation factor is calculated from Equation (15) to increase the comfort function $f_{2}$ in Equation (14), and consequently the fitness evaluation function of each PSO solution according to Equation (16).

In general, in keeping with the initial hypothesis, the integration of user perception variables (humidity and temperature) results in an SHC model with fuzzy comfort than can lead to significant gains in the evaluated scenarios.

### Overview and Other Possible Scenarios

**Figure 10:**
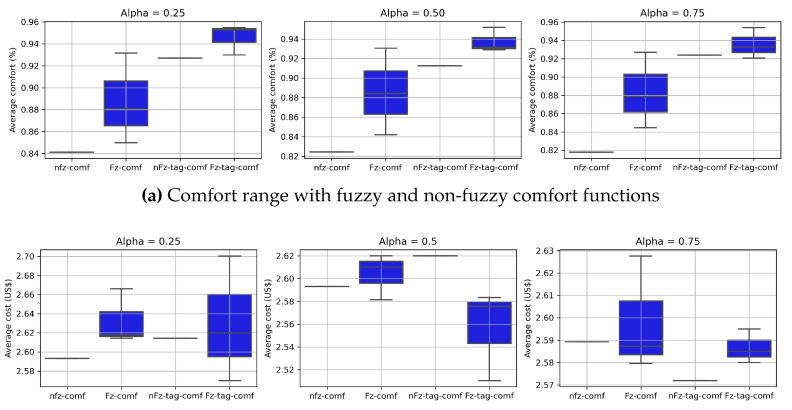
Comfort functions.

Figure 10a,b provides an overview of the tradeoff between comfort and cost for the features originally proposed in [5,25]. Figure 10a shows the user’s comfort gain when the SHC is operated with different comfort functions, while Figure 10b shows the corresponding cost, optimizing the cost–comfort threshold defined for the SHC in each scenario. Thus, it is fair to say that the use of the proposed comfort function allows the SHC to maximize the user’s comfort while including their perception of temperature and humidity and reducing the cost of load scheduling.

Graphs showing the remaining combinations of α with and without considering the user’s comfort perceptions are available on GitHub^®^ at https://github.com/jonathacosta/SmartGrid/tree/main/SCC-SHC/Results/Figures, accessed on 15 February 2023.

## 7. Conclusions

In previous studies, SHCs have been proposed that interact with users’ comfort preferences; however, the dynamics of users’ comfort perceptions have not been studied before. In this context, the present work addresses the problem of modeling user perception of home comfort. Our main objective was to mathematically model the dynamics of user perception of home comfort using fuzzy logic. Based on this model, we analyzed the proposed functions for the housing cost–comfort ratio and compared them with other comfort functions under the same conditions.

The results show that it is more beneficial to use our proposed fz-comfort comfort function in cases when the user asks the SHC to prioritize comfort over financial savings. In other cases, it is more beneficial to use a comfort function that only considers the user’s comfort preferences, i.e., tag-comfort, and not the user’s perceptions. This study makes an initial contribution to modeling users’ comfort perceptions relative to their comfort preferences. A limitation of this study is the context of home comfort, which for the purposes of this study is associated with a single user interacting with the SHC. As a suggestion for future research, there remains a need to analyze user perceptions in multi-user scenarios, such as condominiums or houses, where a single SHC is operated by multiple users.

## Figures and Tables

**Table 1:** Comparison between CT and ToU.

Period	Tariff	CT	ToU
	Mode	(US$/kWh)	(US$/kWh)
00:00 to 16:30	Off-peak	0.136	0.112
16:30 to 17:30	Intermediate	0.136	0.187
17:30 to 20:30	Peak	0.136	0.294
20:30 to 21:30	Intermediate	0.136	0.187
21:30 to 00:00	Off-peak	0.136	0.112

**Table 2:** List of symbols.

Id	Description
*m*	Total amount of schedulable loads
*N*	Total amount of samples
${\bar{P}}_{m}$	Average power vector of *m*-th load
${\hat{P}}_{m}$	Maximum power vector of *m*-th load
$N_{m}$	Duration of the *m*-th load at sampling
$I_{Sm}$	Sample associated with the minimum starting time of the *m*-th load
$I_{Em}$	Sample associated with the maximum end time of the *m*-th load
$I_{Bm}$	Sample associated with the best starting time of the *m*-th load
$I_{Cm}$	Scheduled start time of the *m*-th load.
$C_{Lm}$	Comfort relevance level of *m*-th load
$P_{k}$	Peak limit at *k*-th time instant
*C*	Vector referring to the cost of electrical energy during the period.
$T_{s}$	Consumption sampling rate expressed in minutes
τ	Perception of ambient temperature by the user.
υ	Perception of relative humidity by the user
ω	Comfort relevance level of *m*-th load with user’s perception of τ,υ

**Table 3:** Linguistic variables, terms, and fuzzy domain.

Thermal Perception (τ)		
Linguistic Value	Notation	Domain
Very cold	$t_{1}$	[0.00–0.45]
Cold	$t_{2}$	[0.23–0.68]
Mild	$t_{3}$	[0.40–0.85]
Hot	$t_{4}$	[0.58–1.00]
Very hot	$t_{5}$	[0.65–1.00]
Humidity Perception (υ)		
Linguistic Value	Notation	Domain
Low	$u_{1}$	[0.35–0.50]
Medium	$u_{2}$	[0.40–0.70]
High	$u_{3}$	[0.60–0.75]
Comfort Relevance Level (ω)		
Linguistic Value	Notation	Domain
Low	$c_{1}$	[0.0– 0.4]
Medium	$c_{2}$	[0.2–0.8]
High	$c_{3}$	[0.6–1.0]

**Table 4:** Reference loads in an actual residence.

ID	Load	Cycles	Δt (min)	$\bar{P}$ [kw]	$\hat{P}$ [kw]	Best	Min	Max	$C_{\mathrm{Lm}}$
						Time	Time	Time	
1	Booster pump	1	20	2	3	8 h or 16 h	7 h	17 h	0.1
2	Pool pump	1	120	0.75	1.2	8 h	7 h	17 h	0.1
3	Washing machine	8	10,10, 4, 6, 2, 2, 2, 7	0.13, 0.50, 0.30, 0.26, 0.15, 0.15, 0.15, 0.22	0.70, 0.50, 0.30, 0.26, 0.15, 0.15, 0.15, 0.30	8 h	7 h	17 h	0.5
4	External lighting	1	270	0.3	0.3	18 h	17 h	24 h	0.3
5	Internal lighting	1	270	0.15	0.3	18 h	17 h	23 h	0.7
6	Air conditioning 1	14	[10, 5, 5, …, 5, 5]	[1.3, …, 1.3]	[1.7, 1.3, ⋯, 1.3]	16 h or 20 h	15 h	24 h	1.0
7	Air conditioning 2	7	[30, 20, 5, ⋯, 5, 5]	[2, ⋯, 2]	[2.1, ⋯, 2.1]	20 h	17 h	24 h	1.0
8	Air conditioning 3	1	240	1.1	1.2	20 h	17 h	24 h	1.0
9	Air conditioning 4	7	[10, 10, 5, ⋯, 5]	[0.9, ⋯, 0.9]	[1.1, ⋯, 1.1]	20 h	17 h	24 h	1.0
10	Dis hwashing mac hine	5	5, 10, 15, 5, 10	0.03, 1.76, 0.03, 1.76, 0.03	0.03, 1.76, 0.03, 1.76, 0.03	21 h	18 h	22 h	0.3

**Table 5:** Updating the load relevance from fuzzy inputs.

Id	Load	$C_{Lm}$	τ	υ	ω	Gain
1	Air cond.	1.0	15 °C	40%	0.1639	✓
2	Air cond.	1.0	25 °C	45%	0.4788	✓
3	Air cond.	1.0	38 °C	60%	0.8443	✓

**Table 6:** Comparative results between different fuzzy and non-fuzzy comfort functions using the ToU tariff and α=0.25.

Comfort	nfz-comf		fz-comf		nfz-tag-comf		fz-tag-comf	
ω	∀	0.16	0.56	0.84	∀	0.16	0.56	0.84
$fit_{min}$	1.18	1.20	1.20	1.18	1.20	1.21	1.20	1.21
$fit_{avg}$	1.22	1.23	1.22	1.22	1.23	1.24	1.23	1.23
$fit_{max}$	1.24	1.26	1.25	1.24	1.26	1.27	1.26	1.26
Deviation	0.015	0.016	0.015	0.018	0.016	0.015	0.017	0.013
kW/h	16.4	16.4	16.4	16.4	16.4	16.4	16.4	16.4
US$	2.593	2.666	2.618	2.614	2.614	2.570	2.700	2.619
$Comf_{avg}$	84.13%	93.14%	88.07%	84.99%	92.70%	95.84%	95.28%	92.98%
$t_{avg}$	4.17	3.28	3.36	2.86	3.11	3.10	2.89	2.99

**Table 7:** Comparative results between different fuzzy and non-fuzzy comfort functions using the ToU tariff and α=0.50.

Comfort	nfz-comf		fz-comf		nfz-tag-comf		fz-tag-comf	
ω	∀	0.16	0.56	0.84	∀	0.16	0.56	0.84
$fit_{min}$	1.14	1.15	1.13	1.12	1.13	1.15	1.16	1.16
$fit_{avg}$	1.19	1.21	1.19	1.19	1.12	1.21	1.21	1.22
$fit_{max}$	1.24	1.26	1.24	1.25	1.26	1.25	1.25	1.26
Deviation	0.027	0.029	0.030	0.030	0.035	0.028	0.027	0.026
kW/h	16.4	16.4	16.4	16.4	16.4	16.4	16.4	16.4
US$	2.593	2.581	2.610	2.619	2.619	2.575	2.583	2.510
$Comf_{avg}$	82.43%	93.06%	88.43%	84.19%	91.25%	95.17%	93.13%	92.89%
$t_{avg}$	2.78	2.68	2.84	2.44	2.51	2.82	2.59	2.47

**Table 8:** Comparative results between different fuzzy and non-fuzzy comfort functions using the ToU tariff and α=0.75.

Comfort	nfz-comf		fz-comf		nfz-tag-comf		fz-tag-comf	
ω	∀	0.16	0.56	0.84	∀	0.16	0.56	0.84
$fit_{min}$	1.07	1.05	1.08	1.07	1.05	1.07	1.08	1.11
$fit_{avg}$	1.17	1.16	1.17	1.17	1.18	1.18	1.17	1.17
$fit_{max}$	1.25	1.26	1.26	1.24	1.24	1.25	1.25	1.24
Deviation	0.047	0.049	0.049	0.045	0.043	0.047	0.039	0.039
kW/h	16.4	16.4	16.4	16.4	16.4	16.4	16.4	16.4
US$	2.589	2.628	2.580	2.587	2.572	2.580	2.595	2.585
$Comf_{avg}$	81.80%	92.70%	87.88%	84.47%	92.39%	95.40%	93.27%	92.07%
$t_{avg}$	2.86	2.56	2.52	2.59	2.85	2.78	2.97	2.67

## Data Availability

The data presented in this study are available upon request from the corresponding author.

## Abbreviations

The following abbreviations are used in this manuscript:

DAP	day-ahead price
CPP	critical peak pricing
RTP	real-time price
FP	flat price
CT	conventional tariff
DG	distributed generation
LSSW	load schedule sliding window
LP	linear programming
ILP	integer linear programming
MINLP	mixed-integer nonlinear programming
MILP	mixed-integer linear programming
BPSO	binary particle swarm optimization
TLGO	teacher learning genetic optimization
TLBO	teacher learning-based optimization
GA	genetic algorithm
PSO	particle swarm optimization
FL	fuzzy logic
SA	simulated anneling
ACO	ant colony optimization
MOGWO	multi-objective grey wolf optimization
IoT	internet of things
HIC	home interactive interface
DR	demand response
ToU	time-of-use
SSM	supply-side management
DSM	demand-side management
SG	smart grid
RH	relative humidity
OOP	object-oriented programming
SHC	smart home controller
HVAC	heating, ventilation, and air conditioning
ABNT	Brazilian Association of Technical Standards
ASHRAE	American Society of Heating, Refrigerating, and Air Conditioning Engineers
fz-comf	fuzzy comfort
nfz-comf	non-fuzzy comfort
ESS	energy storage system
EV	electric vehicle
PMV	predicted mean vote
